# Hyperthyroidism in Pregnancy: The Delicate Balance between Too Much or Too Little Antithyroid Drug

**DOI:** 10.3390/jcm10163742

**Published:** 2021-08-23

**Authors:** Monica Livia Gheorghiu, Roxana Georgiana Bors, Ancuta-Augustina Gheorghisan-Galateanu, Anca Lucia Pop, Dragos Cretoiu, Valentin Nicolae Varlas

**Affiliations:** 1 Department of Endocrinology, “Carol Davila” University of Medicine and Pharmacy, 020021 Bucharest, Romania; 2 “C.I. Parhon” National Institute of Endocrinology, 011863 Bucharest, Romania; ancuta.gheorghisan@umfcd.ro; 3 Department of Obstetrics and Gynaecology, Filantropia Clinical Hospital, 011171 Bucharest, Romania; roxana_georgiana.bors@rez.umfcd.ro (R.G.B.); valentin.varlas@umfcd.ro (V.N.V.); 4 Department of Cellular, Molecular Biology and Histology, “Carol Davila” University of Medicine and Pharmacy, 050474 Bucharest, Romania; dragos@cretoiu.ro; 5 Department of Clinical Laboratory, Food Safety, “Carol Davila” University of Medicine and Pharmacy, 020945 Bucharest, Romania; anca.pop@umfcd.ro; 6 Alessandrescu-Rusescu National Institute for Mother and Child Health, Fetal Medicine Excellence Research Center, 020395 Bucharest, Romania; 7 Department of Obstetrics and Gynaecology, “Carol Davila” University of Medicine and Pharmacy, 020021 Bucharest, Romania

**Keywords:** hyperthyroidism, Graves’ disease, pregnancy, antithyroid drug, drug withdrawal, postpartum recurrence, birth defects

## Abstract

Overt hyperthyroidism (HT) during pregnancy is associated with a risk of maternal–fetal complications. Antithyroid drugs (ATD) have a potential risk for teratogenic effects and fetal–neonatal hypothyroidism. This study evaluated ATD treatment and thyroid function control during pregnancy, and pregnancy outcome in women with HT. Patients and methods: A retrospective analysis of 36 single fetus pregnancies in 29 consecutive women (median age 30.3 ± 4.7 years) with HT diagnosed before or during pregnancy; a control group of 39 healthy euthyroid pregnant women was used. Results: Twenty-six women had Graves’ disease (GD, 33 pregnancies), 1 had a hyperfunctioning autonomous nodule, and 2 had gestational transient thyrotoxicosis (GTT). Methimazole (MMI) was administered in 22 pregnancies (78.5%), Propylthiouracil (PTU) in 2 (7.1%), switch from MMI to PTU in 4 (14.2%), no treatment in 8 pregnancies (3 with subclinical HT, 5 euthyroid with previous GD remission before conception). In the 8 pregnancies of GD patients diagnosed during gestation or shortly before (<6 weeks), i.e., with fetal exposure to uncontrolled HT, there was 1 spontaneous abortion at 5 weeks (3.4% of all ATD-treated pregnancies), and 1 premature delivery at 32 weeks with neonatal death in 24 h (3.4%); 1 child had neonatal hyperthyroidism (3.3% of live children in GD women) and a small atrial sept defect (4% of live children in ATD treated women). In women treated more than 6 months until conception (20 pregnancies): (a) median ATD doses were lower than those in women diagnosed shortly before or during pregnancy; (b) ATD was withdrawn in 40% of pregnancies in trimester (T)1, all on MMI < 10 mg/day (relapse in 14.2%), and in up to 55% in T3; (c) TSH level was below normal in 37%, 35% and 22% of pregnancies in T1, T2 and T3 respectively; FT4 was increased in 5.8% (T1) and subnormal in 11.75% in T2 and T3; (d) no fetal birth defects were recorded; one fetal death due to a true umbilical cord knot was registered. Mean birth weight was similar in both ATD-treated and control groups. Hyperthyroidism relapsed postpartum in 83% of GD patients (at median 3 ± 2.6 months). Conclusion: In hyperthyroid women with long-term ATD treatment before conception, drugs could be withdrawn in T1 in 40% of them, the thyroid function control was better, and pregnancy and fetal complications were rarer, compared to women diagnosed during pregnancy. Frequent serum TSH and FT4 monitoring is needed to maintain optimal thyroid function during pregnancy.

## 1. Introduction

Hyperthyroidism occurs due to an inappropriately high synthesis and secretion of thyroid hormones by the thyroid gland and is characterized by a low/suppressed serum level of thyroid-stimulating hormone (TSH), associated with an increased level of peripheral hormones (total and free thyroxine-FT4 and/or total and free triiodothyronine-FT3) in the biochemically overt form of the disease, or with normal FT4 and FT3 levels in subclinical hyperthyroidism [1].

In women, hyperthyroidism is related to menstrual cycle disorders (oligomenorrhea, amenorrhea) and infertility [2]. As a result, a diagnosis of hyperthyroidism during pregnancy is relatively uncommon. The prevalence of overt hyperthyroidism in pregnant women ranges from 0.1 to 0.9%, while subclinical thyrotoxicosis occurs in about 2% [3,4]. Most of these cases are due to Graves’ disease (GD) (90–95%). When the diagnosis of hyperthyroidism is made during the first trimester of pregnancy, the HCG-mediated hyperthyroidism, named gestational transient thyrotoxicosis (GTT), must be taken into account (prevalence 1–11% of pregnancies) [4,5]. In most cases, this is a subclinical hyperthyroidism, which appears after the 6th week of pregnancy due to the physiological rise in HCG secretion; HCG shares structural homology with TSH and stimulates maternal thyroid function, resulting in decreased TSH levels [6]. GTT has a spontaneous resolution by 14–18 weeks of gestation as HCG values decrease [7]. Other causes of thyrotoxicosis (toxic nodular goiters, thyroiditis) are less frequent during pregnancy.

Untreated hyperthyroidism is associated with an increased risk for fetal loss, preterm labor, intrauterine growth restriction, hydrops, congenital malformations in the neonate, and neurobehavioral disorders later in children, as well as maternal complications, such as pregnancy-induced hypertension and maternal congestive heart failure [8,9].

The treatment of hyperthyroidism must consider the etiology, the hormonal changes that occur during pregnancy and influence the course of the disease, and the potential teratogenic effect of the antithyroid drugs (ATD). While treatment is typically not necessary in GTT, it is recommended in GD for overt hyperthyroidism [10,11]. The treatment of choice consists of ATD: Methimazole (MMI), Carbimazole (CMZ), and Propylthiouracil (PTU), which are considered equally effective [7,10,12]. Thioamides inhibit thyroglobulin iodination and iodothyronine synthesis. PTU can also inhibit the conversion of T4 to T3 [10,13]. Alternatively, surgery may be indicated in the second trimester if allergy or intolerance to treatment occurs, but the risk of miscarriage and preterm birth is increased, therefore it is rarely recommended [10,11].

ATD have been associated with several side effects. In pregnant and nonpregnant women rash, urticaria, and arthralgia are more common (1–5%), while severe side effects are rare (0.1–1%): liver toxicity, including fulminant hepatic failure (more often developed on PTU), a lupus-like syndrome, agranulocytosis and, more recently described, a few cases of acute pancreatitis on MMI [11,13,14]. Both MMI and PTU can cross the placenta and induce fetal–neonatal hypothyroidism. When used in early pregnancy, ATD may have teratogenic effects in the fetus [1,14,15] (they are pregnancy category D drugs, i.e., they may be used if the potential benefits outweigh the potential risks for the mother and fetus).

Therefore, the current guidelines recommend using the lowest doses of thioamides that control thyroid function, targeting a maternal serumfree thyroxine (FT4) level at or just above the upper reference limit [10,11]. Whenever possible, notably in women treated and controlled for several months before pregnancy, ATD may be withdrawn in the first trimester [10,11]. Maintaining the delicate balance between too much or too little ATD during pregnancy and breastfeeding is further complicated by the evolution of GD, which may present aggravation in the first trimester, remission during late pregnancy, and relapse during the postpartum period [16,17]. During pregnancy, frequent monitoring of serum TSH and FT4 is recommended to maintain optimal thyroid function [10].

This study aimed to evaluate the rate of thyroid function control on ATD treatment during gestation and the pregnancy outcome in a series of women with hyperthyroidism compared to a control group of healthy women. We also analyzed the differences between women with long-term ATD treatment and symptom control before pregnancy, compared to women with overt hyperthyroidism diagnosed and treated during pregnancy or shortly before pregnancy confirmation (in whom the fetuses have been exposed to uncontrolled maternal hyperthyroidism).

## 2. Patients and Methods

Twenty-nine women diagnosed with current or previous hyperthyroidism and their 36 singleton pregnancies, evaluated consecutively by the authors between 2000 and 2020 in a tertiary care center of Endocrinology and/or a tertiary care center of Obstetrics and Gynecology, were included in a retrospective analysis. The mean age (±standard deviation) was 30.3 ± 4.7 years (22–41) at the diagnosis of pregnancy; 5 women had 2 pregnancies and 1 woman 3 pregnancies.

### 2.1. Study Design

Inclusion criteria. The patients were included if they have been diagnosed with hyperthyroidism (current or cured after ATD treatment), had data regarding the ATD use during pregnancy (dosage, timing), at least 2 evaluations for serum TSH and FT4 during pregnancy, and data regarding the pregnancy outcome.

Exclusion criteria. Pregnant women with a history of hyperthyroidism who had no record on serum TSH and FT4 during pregnancy, no data regarding the ATD use during pregnancy (dosage, timing), or did not consent to register the medical data.

The study focused on the differences regarding ATD dose, ATD withdrawal (early or late during pregnancy), thyroid function control during and after pregnancy, pregnancy outcome (at term, spontaneous abortions, premature delivery, type of delivery), and children outcome (live children, birth weight, birth defects, fetal or neonatal hypo- or hyperthyroidism) in 16 women (20 pregnancies) with long-term ATD treatment (more than 6 months) and symptom control before pregnancy, compared with 7 women (8 pregnancies) with overt hyperthyroidism diagnosed and treated during pregnancy or shortly before pregnancy confirmation (less than 6 weeks). In addition, data for 3 untreated subclinical hyperthyroid women and for 5 currently euthyroid women after previous GD remission were presented, but not included in the statistical analysis. A control group of 39 pregnant women with normal thyroid function (evaluated before delivery) and 39 single fetus pregnancies, who delivered at term, was compared for pregnancy outcome. They were randomly recruited from the Filantropia Hospital; mean age was 27 ± 4.1 years (19–37); mean serum TSH during pregnancy (before delivery) was 1.85 ± 0.88 mIU/L (range 0.36–3.95 mIU/L), normal range 0.35–4.5 mIU/L.

### 2.2. Data Collection

#### 2.2.1. Evaluation of the Thyroid Function and Treatment

Thyroid function was evaluated with various commercial assays. Serum TSH was considered low if below 0.1 mIU/L and high if >2.5 mIU/L in trimester 1 or >3.5 mIU/L in trimester 2 and 3; FT4 was compared with the non-pregnant normal values; good thyroid function control during pregnancy was defined by a normal serum FT4. No local references for pregnancy were available either for TSH, FT4, or T3. Data regarding treatment with ATD, type of treatment, dose, duration, drug switch, drug withdrawal, side effects were retrieved from medical files.

#### 2.2.2. Evaluation of the Pregnancy Outcome

Data regarding pregnancy duration, outcome, complications of mother and fetus, type of delivery, children’s sex, weight, APGAR score, birth defects, or other complications in the neonates were collected from the files. The trimesters of pregnancy are abbreviated as T1, T2, T3. The patients have signed an informed consent to use their medical data for scientific research, approved by the institutional ethics committee.

### 2.3. Statistics

Normally distributed data are presented as mean ± standard deviation (SD) and compared using *t*-test. Data with non-normal distribution are presented as median ± standard deviation (range) and compared with ANOVA test. Statistical processing was performed using SPSS 22.0 software package. Results with *p* < 0.05 were considered statistically significant.

## 3. Results

### 3.1. Demographics

The main characteristics of the 29 patients (36 pregnancies) are described in Table 1 and Table 2.

### 3.2. Etiology of Hyperthyroidism

Graves’ disease (GD) was diagnosed in 26 out of 29, 89.6% pregnant women (33 out of 36 pregnancies, 91.6%), hyperfunctioning autonomous nodule in 1 pregnancy (3.4% of women, 2.7% of pregnancies), and transient gestational thyrotoxicosis (GTT) in 2 women (6.8% of women, 5.5% of pregnancies).

The diagnosis of GD was based on suppressed TSH, high total thyroxine (T4)/FT4 or/and T3, and increased TSH receptor antibodies-TRAb (>1.5–1.75 IU/L, depending on the assay) or antithyroid peroxidase antibodies (TPOAb) when TRAb was not available. A goiter was palpable in 20 GD patients (no data in the other 6); Graves’ ophthalmopathy was recorded in 7 out of 22 GD patients (31.8%): mild in 6 women, moderate in one.

The patient with autonomous nodule had a multinodular goiter with a dominant 2.5 cm left lobe nodule with increased I^131^ uptake, and normal TRAb and TPOAb. The two patients with GTT had normal values of TSH and FT4 before pregnancy, suppressed TSH, normal or borderline high FT4 and normal TRAb in the first trimester, and again normal TSH and FT4 after 4–6 weeks (in the 2nd trimester). One had a small diffuse goiter.

### 3.3. Treatment with ATD

Sixteen out of 29 women (20 pregnancies) had been treated with ATD for more than 6 months until pregnancy (up to 5 years). At the diagnosis of pregnancy 1 woman had mildly symptomatic overt hyperthyroidism, 2 had subclinical hyperthyroidism, and the others had normal thyroid function.

In the other 8 pregnancies, the treatment was either started shortly before the diagnosis of pregnancy (less than 6 weeks, n = 2), or during pregnancy, in week 12–25, median week 16. All were hyperthyroid in pregnancy (Table 2 and Table 3).

*ATD type* (Table 1 and Table 3). Most of the treated patients received Methimazole (MMI)—in 22 out of 28 treated pregnancies (78.5%), Propylthiouracil (PTU) in 2 (7.1%), switch from MMI to PTU in 4 pregnancies (14.2%); no treatment was administered in 8 pregnancies (2 with GTT, 1 with a chronic subclinical GD, 5 in previous GD remission, with euthyroidism maintained without ATD before conception); of note, in Romania PTU is not commercially available.

*ATD dosage* (Table 2 and Table 3). Pooled data using a 20:1 equivalence ratio for PTU to MMI showed that in women with previous long-term ATD treatment, the median doses at the diagnosis of pregnancy, i.e., 5 mg/day (range: 2.5 mg every other day—15 mg/day) were lower than those administered in women diagnosed de novo or shortly before pregnancy: 17.5 mg/day (range 5–45 mg/day), *p* < 0.05. However, the difference between groups was no longer significant in the 3rd trimester, showing the potential for rapidly decreasing doses even in women diagnosed during pregnancy.

*ATD withdrawal* was recorded in T1 in 8 (40%) of the pregnancies of long-term treated women (controlled on MMI doses ranging from <2.5 mg up to 10 mg/day) and in up to 55% of pregnancies in T3 (11/20 pregnancies). The withdrawal was also possible during T3 in 3 of 7 (42.8%) patients diagnosed shortly before or during pregnancy (*p* = NS compared to the long-term treated group). In 2 patients who withdrew ATD, treatment was restarted due to hyperthyroidism relapse: in 1 of 8 patients after withdrawal in T1 (14.2%) and in 1 patient after withdrawal in T2 (out of 5, 20%).

### 3.4. Control of Thyroid Function during Pregnancy

In patients on long-term ATD before pregnancy, the maternal TSH level was below normal in 37% of pregnancies in T1, in 35% in T2, and in 22% in T3; high FT4 was recorded in 5.8% of pregnancies (in T1 only), and subnormal FT4 was noted in 11.7% of pregnancies in T2 and in T3 (Table 2 and Table 3). However, in more than 80% of cases, FT4 levels were below the upper 1/3 of the normal non-pregnant range. In patients who started ATD shortly before or during pregnancy (*n* = 8), hyperthyroidism persisted in 2, subclinical hyperthyroidism in 4, euthyroidism was followed by low FT4 in one, no data after ATD was started is available for the last patient. Overall, thyroid control during pregnancy was significantly better in long-term treated women than in those diagnosed shortly after or during pregnancy, notably in the first two trimesters (Table 3).

Two patients diagnosed with GTT did not receive ATD treatment and normalized their thyroid function at the beginning of T2. The untreated subclinical GD spontaneously normalized in T2.

Evolution in the 5 patients considered in GD clinical remission, without treatment before and during pregnancy: a tendency towards subclinical hypothyroidism/hypothyroxinemia developed during pregnancy, and LT4 was started in 2 patients, the others remained euthyroid during gestation.

### 3.5. Postpartum Recurrence/Relapse/Aggravation of Hyperthyroidism

Postpartum recurrence/aggravation of hyperthyroidism was noted in 20 out of 24 (83.3%) pregnancies of treated women, all recorded in patients with GD (at median 3 ± 2.6 months, range 1–10 months); recurrence was also noted in 3 of 5 (60%) patients with previous remission of the hyperthyroidism before pregnancy (all having elevated TRAb levels postpartum (Table 1 and Table 2).

### 3.6. Pregnancy Outcome

Details are shown in Table 4.

There were 31 live children out of 34 (91.1%, 2 patients were lost to follow-up); one spontaneous abortion at 5 weeks (3.4% of pregnancies in women with hyperthyroidism), and one premature delivery (3.4%) at 32 weeks, with perinatal death within a few hours (respiratory distress) (3.4%). These were recorded in 2 of the 8 patients with GD diagnosed shortly before or during pregnancy (25%). One fetal death at 40 weeks due to a true umbilical cord knot (3.4% of all pregnancies) was recorded in the 20 patients with long-term ATD before pregnancy (5%).

No goiter was recorded in the fetuses or live neonates; only 1 case of neonatal hyperthyroidism (3.3% of 30 live children from GD women) and 1 birth defect (4% of children of ATD treated women), i.e., a small atrial sept defect—ostium secundum, with mild systolic murmur (II/VI), were noted in a child born to a GD patient diagnosed and treated with MMI from the beginning of T3 onward. The follow-up was longer than 2 years in 13 out of 25 children (52%) from women with ATD treatment during pregnancy.

No neonatal hypothyroidism was declared by the patients (however, data on this subject were missing in most of the patient’s files).

**Figure 1:**
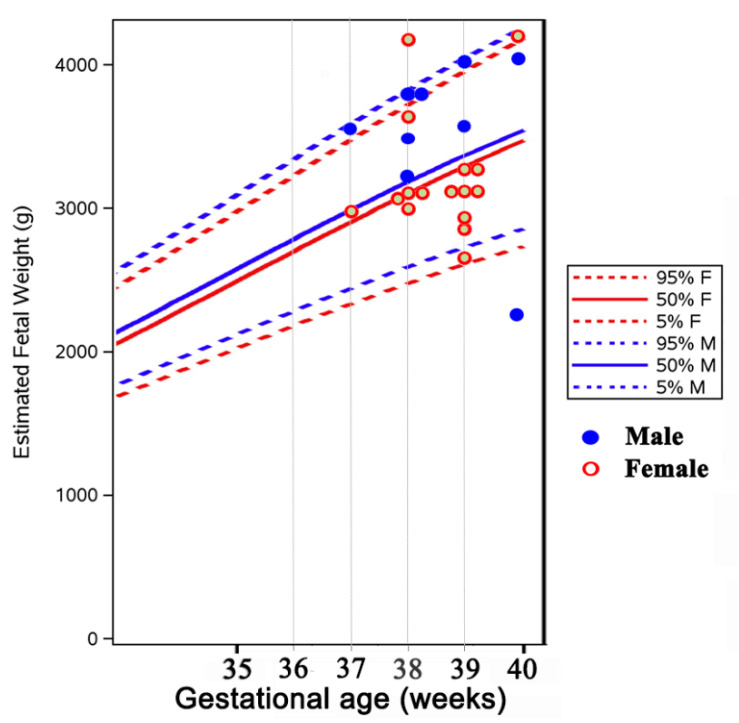
Distribution of birth weight in newborns from mothers with hyperthyroidism (plotted on the WHO fetal growth chart [18].

**Figure 2:**
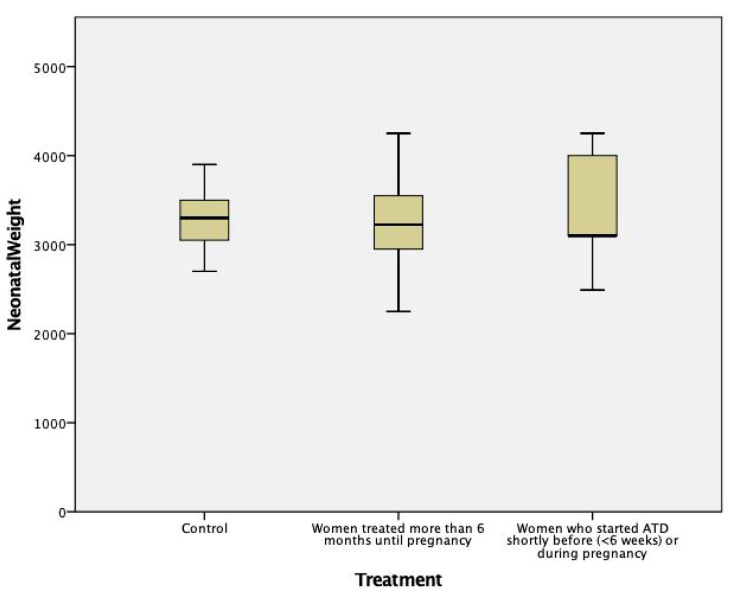
Newborn’s weight in mothers treated with ATD compared to healthy controls (*p* = NS between groups).

The gestational age and birth weight of children born to ATD treated women were not significantly different from those of children born to normal control women, either for the whole group (Table 4, Figure 1 and Figure 2) or separated by sex (data not shown). They were also similar in children born to women with long-term ATD treatment, compared to those from women who started treatment shortly before or during pregnancy. No difference was noted in the percent of cesarean (C)-sections in treated versus control women (most C-sections were performed for obstetrical reasons unrelated to hyperthyroidism, except moderate exophthalmos).

## 4. Discussion

Uncontrolled hyperthyroidism during pregnancy may adversely affect both mother and child, therefore, it should be diagnosed, treated, and ideally cured before gestation [3,10,11]. However, in real life, this is not possible in all cases. Women with symptomatic, overt hyperthyroidism require treatment with antithyroid drugs (ATD), while in those with subclinical hyperthyroidism or GTT, treatment with ATD is not recommended [10,11]. The lowest doses of ATD should be used to control the thyroid function (aiming a maternal serum FT4 level at or just above the upper reference limit), avoid fetal hyper—or hypothyroidism, and minimize the risk of ATD-induced teratogenic effects [10,11].

Our study evaluated the rate of thyroid function control on ATD treatment during gestation and the pregnancy outcome in women with hyperthyroidism compared to a control group of healthy women. We also analyzed the differences between women with long-term ATD treatment and symptom control before pregnancy, compared to women with overt hyperthyroidism diagnosed and treated during pregnancy or shortly before pregnancy confirmation (in whom the fetuses have been exposed to uncontrolled maternal hyperthyroidism).

The diagnosis and treatment of hyperthyroidism during pregnancy meet specific challenges.

### 4.1. Difficulties in the Diagnosis of Hyperthyroidism during Pregnancy

It is essential to identify the underlying etiology of thyrotoxicosis in pregnancy to recommend the appropriate treatment. Graves’ disease (GD) and the first-trimester HCG-mediated hyperthyroidism (GTT) are the most common causes of thyrotoxicosis during pregnancy [3,4]. Approximately 90–95% of hyperthyroid pregnant women have GD [4]—90.3% in our study and 1–11% have GTT (6.4% in our study). Other less frequent causes of thyrotoxicosis during pregnancy include toxic multinodular goiter and toxic adenoma (in <5% of cases, as in our series) and subacute thyroiditis (more frequent in the postpartum period) [3,4].

The signs and symptoms of hyperthyroidism may be confounded with physiological changes of pregnancy: tachycardia, heat intolerance, anxiety, emotional lability, diaphoresis, fatigue, gastrointestinal symptoms such as nausea, vomiting, and diarrhea (which may be confounded with hyperemesis gravidarum). More specific signs are hand tremor, weight loss despite a normal or increased appetite [19], exophthalmos—suggesting the diagnosis of GD, signs, and symptoms of congestive heart failure [11,19].

The biochemical diagnosis is hindered by the physiological adaptive changes which occur since early pregnancy and influence the maternal thyroid function and hormonal tests. The early HCG rise induces a decrease in serum TSH, a serum HCG concentration of 10,000 UI/L being associated with a 0.1 mUI/L decrease of TSH serum concentration [20,21]. Between weeks 7 and 11 of gestation, a TSH < 0.1 mIU/L occurs in about 5% of normal women, with levels being completely suppressed in 0.5 to 1% [22], usually at HCG levels of more than 50,000 mIU/mL. There is an increase in the synthesis of TBG (thyroxine-binding globulin) caused by the high serum levels of estrogen, leading to an increase in total T4 and T3 levels, but usually not in the free T4 and T3 [6,23].

The increased TBG values and hemodilution may influence the measurement of free T4 by indirect analog immunoassays [24]. In pregnancy weeks 9–12, the FT4 upper reference limit may be slightly higher (~5%) than the non-pregnancy reference limit, while in the second, and especially in the third trimester is lower than non-pregnancy values [22,23]. These changes should be considered when assessing the diagnosis and the ATD control of the thyroid function. The use of local trimester-specific and assay-specific reference ranges for TSH and free T4 is recommended [10,11], but they were not available for the patients in our series.

For the differential diagnosis of first-trimester hyperthyroidism, the evidence of increased serum TRAb levels, increased T3 or T3/T4 ratio, and the signs of orbitopathy on clinical examination are characteristic for GD and differentiate GD from GTT [7,10,11]. The thyroid gland volume may be increased, as it was in the GD women in our series. In contrast with GTT, GD pregnant women typically require treatment.

GTT is usually associated with mild thyrotoxic symptoms, which spontaneously subside by 14–18 weeks of gestation as HCG values decrease [7]. The thyroid gland is not enlarged. HCG-mediated hyperthyroidism is more frequent in the circumstances associated with high serum concentrations of HCG, such as multiple pregnancies, hyperemesis gravidarum (loss of 5% body weight, dehydration, and ketonuria), gestational trophoblastic disease [5,10], or rare mutations in the TSH receptor [25]. Levels of HCG correlate with the degree of nausea and the severity of hyperthyroidism [15].

GTT is associated with a favorable outcome of the pregnancy and usually does not require antithyroid treatment [5,7] (which was not prescribed in our 2 women with subclinical GTT).

Endemic goiter due to iodine deficiency may pose difficulties in the diagnosis of gestational hyperthyroidism. Iodine clearance is two times higher during pregnancy due to the increased renal blood flow and glomerular filtration rate. The thyroid gland volume increases by up to 25% during pregnancy in an attempt to adapt to the increased thyroid hormone production required during pregnancy [6]. As a result, goiter during gestation is more frequent in women from iodine deficiency regions [6] and could produce difficulties in identifying the cause of concomitant hyperthyroidism (as it was the case, in our study, in 1 GD patient with a thyroid nodule and a GTT patient with goiter). Both iodine excess and deficiency are harmful and impair fetal development [26]. In our country, in which iodine deficiency is present on 2/3 of the territory, we have previously shown higher cord blood levels of TSH and lower levels of T4 in newborns from iodine-deficient areas compared to those from iodine sufficient regions [27]. An increased iodine intake (250 mcg of iodine daily) is recommended in pregnant and lactating women by the World Health Organization [10]). After introducing the universal salt iodization in Romania in 2002, median urinary iodine concentration normalized in schoolchildren and improved in pregnant women from iodine-deficient areas [28,29].

### 4.2. Treatment with ATD and Thyroid Function Control in Pregnant Women with Hyperthyroidism

The goal of the treatment in pregnant women with hyperthyroidism is to provide an excellent fetal and maternal outcome by preventing the complications induced by uncontrolled maternal hyperthyroidism, as well as the development of maternal or fetal hypothyroidism [7,10].

Therapeutic strategies depend on the cause and severity of the disease, timing of the diagnosis in relation to pregnancy (before or during early or advanced pregnancy) and should take into account the risk of side effects [7,10,12]. Thioamides are the mainstay of the treatment of hyperthyroidism during pregnancy.

In our series, women diagnosed before pregnancy were counseled regarding the risks and benefits of every treatment option (surgery, radioiodine, or medical therapy). According to the current guidelines, the preferred ATD for those planning a pregnancy and those diagnosed de novo during early pregnancy is PTU, due to the lower reported prevalence of birth defects [10,11]. Unfortunately, PTU is not commercially available in our country. Only 21% of the patients (all from the long-term treated group) were able to shift to PTU before or after pregnancy was diagnosed (as currently recommended [10,11]), the others being treated with MMI. However, it should be noted that data from large trials and recent meta-analyses have shown that shifting MMI to PTU in early pregnancy (usually in a 1:20 ratio) was still associated with an increased risk for birth defects [1,30,31].

From the beginning of the second trimester onwards, treatment with MMI is preferred, in view of the potential PTU—induced liver toxicity, but PTU may also be continued [10,11]. In our series of patients, women who switched from MMI to PTU continued with it until delivery, with no maternal adverse effects.

To avoid ATD treatment during the first trimester, in women who are controlled on low-dose ATD [MMI ≤ 5–10 mg/day or PTU ≤ 100–200 mg/day], who have already been treated for at least 6 months (or are euthyroid on a stable dose checked 2 months apart [11]), who do not have large goiters or TRAb > 3 times the upper limit of normal), ETA and ATA guidelines suggest withdrawing ATD as soon as pregnancy is diagnosed [10,11], closely monitoring symptoms and thyroid function every 1 to 2 weeks [7,12]. In our series, ATD was withdrawn in the first trimester in 40% of women treated for at least 6 months before pregnancy (these fulfilled the above criteria). Hyperthyroidism relapsed only in 1 patient (14.2%) in about 8 weeks, and ATD was resumed. Reviewing the data, a few other patients may have been good candidates for this strategy, which was not recommended at that time. More studies are needed to evaluate the benefits and risks of this procedure. Of note, the recent guidelines of the Association of Canadian Obstetrics and Gynecology do not mention this strategy [32].

In women with newly diagnosed GD during gestation, the guidelines recommend using the lowest doses of thioamides that control thyroid function (usually PTU 50 mg 2 or 3 times daily, MMI 5–10 mg daily, or CMZ 5–15 mg daily) [10,11]. This was possible in most of our patients, except in those with moderate to severe hyperthyroidism diagnosed during pregnancy or shortly before, in whom the starting doses have been significantly higher (10–45 mg/day) (Table 3).

To maintain an optimal control of the thyroid function in both mother and fetus, frequent monitoring of TSH, free T4, and/or total T4 or T3 level is recommended throughout pregnancy (every 4 weeks in women on stable doses of ATD, or even more frequently when dose adjustments are made) [10,11,12]. This was not possible in all our cases in real life, usually due to patient non-compliance or incomplete available data—a recognized limitation of our retrospective analysis. However, a good control of the thyroid function during pregnancy was obtained in about 88% of women treated more than 6 months before conception, compared to 60–70% in women diagnosed shortly before or during gestation (Table 3). Higher (but not statistically significant) levels of TRAb were found in the latter group (mean 12.2 IU/L) compared to those in the long-term treated group (mean 3.8 IU/L, *p* = 0.21).

GD patients may enter remission during late pregnancy (due to the progressive decrease of TRAb titers), and ATD may be withdrawn [17]. In our series, 55% of the long-term treated pregnant women and 42.8% of those diagnosed during pregnancy were treatment-free in the last trimester. However, GD frequently relapses during the postpartum period, which indeed was the case in 83.3% of our patients in the following 10 months. Relapse of thyrotoxicosis was also noted in 3 of the 5 women (60%) considered in previous stable remission/cure of GD (Table 1). In this case, the prevalence may be overestimated, patients with recurrence being more prone to present to the endocrinologist. The differential diagnosis with postpartum thyroiditis may be challenging since these patients may have persistent TRAb, and radioiodine uptake is not readily performed in breastfeeding women. Regular monitoring of TSH and FT4 is thus warranted in the postpartum period, notably in the first 3 to 12 months [10,11]. Breastfeeding is possible in women treated with MMI < 20 mg/day or PTU < 250 mg/day, taken in divided doses after feeding [11,33] and we recommend it.

### 4.3. Fetal and Neonatal Outcomes

*Intrauterine growth restriction* was reported in women with hyperthyroidism; it is caused by direct effects of the excess thyroid hormones and by the associated preeclampsia [34]. In our study, only 1 child had a birth weight below the 5th percentile of normal children (4.1% of the treated pregnancies); he was born to a mother treated with MMI during the whole pregnancy (10 to 5 mg/day). Mean birth weight did not differ significantly in children born to women diagnosed de novo during pregnancy, in those from women treated with ATD since before pregnancy, and in those from normal control women (Table 4, Figure 2).

*Birth defects* were recorded in approximately 4–9% of pregnancies with MMI exposure and in 1.9–8% of pregnancies with PTU exposure in several but not all studies [1,13,30,35,36]. A recent meta-analysis including 6,212,322 pregnancies and 388,976 birth defects from 7 cohort studies and 1 case-control study has calculated even lower adjusted risks of having congenital anomalies related to ATD compared to the unexposed population [31]. The excess risk for any and major birth defects per 1000, respectively, was 10.2 and 1.3 for PTU, 17.8 and 2.3 for MMI/CMZ, 32.5 and 4.1 for both MMI/CMZ and PTU, 9.6 and 1.2 for untreated hyperthyroidism [31].

MMI and CMZ -associated defects include aplasia cutis, facial dysmorphism, or more severe, albeit rare, defects in the so-called MMI/CBZ embryopathy: choanal or esophageal atresia, omphalocele, omphalomesenteric duct anomalies, and other abdominal wall defects and ventricular septal defects [1,30,35].

Congenital defects associated with PTU use are milder, include face and neck cysts and urinary tract defects in males, and may be revealed later, 1–2 years after birth [35]. There is not a clear association between the PTU dose and the occurrence of birth defects, although this seems more convincing for MMI [1]. The most sensitive period for teratogenesis is between 6 and 10 weeks of pregnancy [37]. Therefore, PTU is preferred over MMI during the pre-pregnancy period and through the first trimester, while the use of “block and replace” treatment with high doses of ATD and L-thyroxine is not recommended during pregnancy [10,11].

In our series, in which about half of the children have been followed–up for more than 2 years, there were no serious birth defects described in the 25 live neonates from women treated with ATD during pregnancy, only 1 small atrial sept defect (4% of live children in ATD treated women). The median dose of MMI during early pregnancy was usually low, 5 mg/day in women treated for at least 6 months before pregnancy, and higher, around 20 mg/day in women treated less than 6 weeks before conception.

Taking into account the small risk of birth defects, we agree with the authors of a recent position paper of several Italian Societies of Endocrinology and Obstetrics-Gynecology [13], that in MMI-treated women of reproductive age, contraceptive methods are not routinely necessary (they should be offered until disease control is obtained). In women with an unplanned pregnancy, notably when they are well controlled on low ATD doses, therapeutic abortion is not warranted.

*ATD-induced fetal hypothyroidism* is another risk of ATD treatment which should be avoided by using the lowest possible dose to keep the maternal serum-free T4 at or just above the upper limit of normal [7,10,11,12]. Maternal thyroid hormones are essential for the fetal central nervous system development during the first trimester when the fetal thyroid is not functional [38]. The fetal thyroid begins to secrete thyroid hormones from about week 16 of gestation and is more sensitive to ATD than the maternal thyroid [39]. Low thyroid function after birth is encountered in one-half of neonates whose mothers were treated with PTU or MMI during pregnancy and who had normal serum T4 concentrations [39].

High, as well as low, maternal free T4 levels have been associated with adverse effects on child IQ at 6 years of age [9], and maternal hyperthyroidism may be associated with higher risks for seizures and attention deficit hyperactivity symptoms later in children [40].

In our series, there were no cases with neonatal hypothyroidism recorded. The data are limited because the neonatal TSH screening program for congenital hypothyroidism in Romania has been generalized since 2011, and only children with dry-spot TSH ≥ 17 mIU/L are recalled [41].

### 4.4. Fetal and Neonatal Hyperthyroidism

GD with high maternal TRAb titers (>3 times the upper limit of normal in the second or third trimester) increases the risk for fetal or neonatal hyperthyroidism by placental transfer, which begins by the 20th week and peaks by the 30th week [12,42,43]. Neonatal hyperthyroidism would occur in 1 to 5% of infants of women with a history of GD, even if the mother had undergone thyroidectomy or radioiodine ablation [21]. The fetus should be monitored by ultrasound for signs of hyperthyroidism (sustained tachycardia > 160 bpm, goiter, intra-uterine growth restriction, advanced bone age, craniosynostosis, nonimmune hydrops) or hypothyroidism (goiter, retarded bone age) [10,11,44,45]. We had only 1 case of neonatal hyperthyroidism (3.3% of live children born to GD women) in a patient with very high TRAb levels (36 IU/L) diagnosed with overt GD in the third trimester. TRAb monitoring was not regular in every patient; however, more frequent ultrasound monitoring of the fetus was undertaken, especially in women who still needed treatment or had uncontrolled hyperthyroidism in late pregnancy, in line with the ACOG guidelines [32].

## 5. Conclusions

Few studies have analyzed the management of hyperthyroid pregnant patients and the outcomes for the mother and child. In this small retrospective study, we demonstrated that most women with hyperthyroidism treated with antithyroid drugs (ATD, usually methimazole)—had normal pregnancies. Better thyroid function control and lower ATD doses were used, with better pregnancy outcome, in women with long-term treatment before conception (months), as compared to women diagnosed and treated during pregnancy or just a few weeks before, in whom the fetus was exposed to uncontrolled maternal hyperthyroidism and higher ATD doses. ATD withdrawal during early pregnancy to avoid the sensitive period to teratogenesis was possible in 40% of the long-term treated patients (who were well controlled on low ATD doses); it was also possible in about half of the patients with Graves’ disease during late pregnancy. Frequent TSH and FT4 monitoring is needed to obtain good control of the thyroid function, notably in patients diagnosed during pregnancy, as well as in GD patients after delivery (in whom recurrence or aggravation of hyperthyroidism occurred in 83% of cases). Birth defects associated with ATD were mild and rare in our small patient series (1 in 25 children, 4%). We recommend a multidisciplinary approach (endocrinologist + obstetrician) to reduce fetal–maternal risks in hyperthyroid women. Extensive prospective studies are still needed to evaluate the currently recommended management strategies in women with hyperthyroidism during pregnancy.

## Figures and Tables

**Table 1:** General characteristics of pregnant women with hyperthyroidism.

Patients	*n* = 29 Women and 36 Single Fetus Pregnancies
Age at pregnancy, years, mean ± SD	30.3 ± 4.7 (range 22–41)
Etiology of hyperthyroidism	Graves’ disease (GD): *n* = 26 (33 pregnancies) Hyperfunctioning autonomous nodule: *n* = 1 Transient gestational thyrotoxicosis: *n* = 2
Graves ophthalmopathy	*n* = 7 (6 mild, 1 moderate) (7/22 evaluable GD women, 31.8%)
Treatment with antithyroid drugs (ATD) during pregnancy	Treatment: 28 pregnancies Methimazole (M): 22 pregnancies (78.5% of treated women) Propylthiouracil (P): 2 pregnancies (7.1%) Switch from M to P: 4 pregnancies (14.2%) No treatment: 8 pregnancies (2 with GTT, 1 subclinical GD, 5 euthyroid in previous GD remission before conception)
Treatment withdrawal during pregnancy	16 out of 28 treated pregnancies (57.1%), withdrawal maintained until delivery in 14 out of 28 pregnancies (50%) - At pregnancy diagnosis: *n* = 8 (resumed in 1–14.2%) - During 2nd trimester: *n* = 5 - During 3rd trimester: *n* = 3
Recurrence or aggravation of thyrotoxicosis after delivery	20 out of 24 pregnancies in ATD treated women (83.3%, all with GD); 3 out of 5 women with GD remission and no treatment be fore & after conception (60%) Median interval ± SD until recurrence/aggravation (range): 3 ± 2.6 months (range 1–10 months)

Legend: GTT—gestational transient HCG-mediated thyrotoxicosis; GD—Graves’ disease.

**Table 2:** Synopsis of women with hyperthyroidism, monitored during pregnancy.

No./Age (Years)	Before Pregnancy		First Trimester	Second Trimester	Third Trimester		Postpartum H. Recurrence/Aggravation (Months)
	Dose/Thyroid Status	TRAb (IU/mL)	Dose/Thyroid Status	Dose/Thyroid Status	Dose/Thyroid Status	Pregnancy Outcome/Delivery	
I. Hyperthyroid women treated with ATD more than 6 months until pregnancy							
1.1 (27)	M 2.5/2d/N	8.5→<0.3	-/N	-/N	-/N	girl 3300 g, A8, C-section 39w	4
2 * (36)	M 2.5/2d/N	n.a	M 2.5/2d/N	M 2.5/2d/N	-/N	girl, 2950 g, C-section 37w	2
1.2 (30)	M 2.5/N	5.15	M 2.5/2d/N/SH	M 2.5/SH/hT/N	-/N	girl, 3300 g, A9, C-section 39w	3
3 (30)	M 2.5/N	n.a	M 2.5/N	M 2.5/N	M 2.5/N	girl, 2950 g, A7, C-section 38	No on M 10
4 (37)	M 5/N	1.66	-/N	-/N	-/N	boy, 3500 g, C-section 36w	4
5.1 (25)	M 5/N	n.a	-/N	-/N	-/N	Intrauterine death ^&^, C-section 40w	7
5.2 (26)	M 5/N	n.a	-/N	-/N	-/N	girl, 2800 g, A9, C-section 38w	7–8
5.3 (29)	M 5/N	6.72	-/N	-/N	-/N	boy, 3750 g, A10, C-section 39w	7
6 (31)	M 5/N	1.37	-/N	-/N	-/hT/N	girl, 3150 g, A9/10, Vaginal 39w	n.a
7.2 (31)	M 5/N	n.a	M 2.5–5/N	-/N	-/N	boy 3500 g, A10, C-section 39w	1
8.1 (27)	M 5/N	n.a	P 50/SH/N	P 25–37.5/N	P 12.5/2d/N	boy, Vaginal at term	3
9 (26)	M 10/N	2.07	-/N	-/n.a	-/N	girl, 2750 g, A10, C-section 39w	3 (SH)
10 (34)	M 10/N	0.31	M 10/N/H	M 7.5–10/SH/N	M 2.5–5/N	girl, 3000 g, A10, Vaginal 28w	No on M 5
11 (31)	M 10/H	n.a	M 10/N	M 10/N	M 10/n.a	boy, 3700 g, A9-10, C- section	n.a
12 (29)	M 10/N	n.a	M 10/N	M 6.25–7.5 hT/N	M 5–6.25/hT/N	boy, 2250 g, A9, C-section 40w	No on M 5
13 (23)	M 10/n.a	n.a	P 150–250/SH	P 150/SH	P 150/SH	girl, 2700 g, A10, C-section 39w	3
14 (31)	M 10/n.a	3.69 ^	M 10/n.a	P 50–100/SH	P 50/SH	girl, 4250 g, A9-10, C-section	n.a
15.1 (36)	M 10/N	n.a	P 100–150/H	P 100–150/N	P 100/N	boy, 3700 g, A10, C-section 38w	3
15.2 (38)	P 200/SH	n.a	P 50–150/SH	P 50/SH	-/SH	girl, 3600 g, A10, C-section 38w	1
16 (22)	P 300/SH	13.73	P 300/n.a	P 150/SH	P 150/SH	girl, 3140 g, A9, C-section 39w	3
II. Overt hyperthyroid women diagnosed shortly before or during pregnancy							
IIa. Women treated with ATD less than 6 weeks until pregnancy							
17 (26)	M 20 1 month/H	4.19	M 30/H	M 10/SH/N	-/N	girl, 4250 g, A9, Vaginal 40w	1.5
18.1 (25)	M 40–20 1 month/H	n.a	M 15/SH			Spontaneous abortion at 5w	10
IIb. Women who started ATD during pregnancy							
19 (26)	M 6 months, stop 3.5 years	n.a	-	M 45/H	M 30/H	boy; neonate death; Vaginal PD 32w	n.a
20 (29)	-	24.5 ^	-	M 2.5–30/H/N	M2.5/2d/-/hT	girl, 3100 g, C-section	10 (not tested before)
7.1 (29)	-	5.44 ^	-	M 5–10/H	M 5/SH	girl, 3100 g, A9, C-section 39w	3
21 (30)	-	1.89; 1.57 postP	M 10–7.5/H	M 2.5–5/H-SH	-/SH	girl, 2490 g, Vaginal 39w	2 (borderline SH), then N
18.2 (26)	M 7 months, stop 5 months	1.56 ^	-/H	M 5/H	M 5/n.a	boy, 4000 g, A9, Vaginal 40w	4
22 (40)	-	36	-	-	M 15–20/H	girl, 3170, A9, C-section 38w	Stable mild H at 3 weeks
III. Women with hyperthyroidism not treated with ATD during pregnancy							
IIIa. Transient gestational thyrotoxicosis							
23 (33)	-/N	0.41	-/SH	-/N	-/n.a	girl, Vaginal 39w	No
24 (30)	-/N	0.28 ^	-H/SH	-/N	-/n.a	n.a	n.a
IIIb. GD with subclinical hyperthyroidism							
25 (33)	M 4 months, stop 2.5 years/SH	5.55	-/SH	-/N	-/N	girl **, n.a	n.a
IV. Women with hyperthyroidism in remission after previous ATD treatment (euthyroid, not treated during pregnancy)							
26 (35)	M 9 months, stop 4 years/N	13.2 postP	-/N	-/N	-/N	girl 3150 g, A9/9, Vaginal	3
27 (32)	M 4 years, stop 3 years/N	10.3 postP	-/N	-/N	-/N	boy, 3150 g, A10, C-section 39w	4
28 (26)	M 9 months, stop 3 months/N	2.41	-/N	-/N	-/N	boy, 3260 g, A8, C-section at term	No
8.2 (31)	M 4 years, stop 1 year/N	1.5	-/N	LT4-12.5-37.5/Sh/N	LT4-50/Sh	Child, Vaginal at term	No
29 (41)	M 1 year, stop 1.3 years/N	3.88→0.3 1.94 postP	-/n.a	LT4-25-37.5/N-hT	LT4 50-62.5/hT	Child, C-section at term	6.5

Legend. All women have been diagnosed with Graves’ disease (GD), except patient 12 who had an autonomous hyperfunctioning nodule and 2 patients (23 & 24) with transient gestational hyperthyroidism in the first trimester; TRAb = thyrotropin receptor antibodies; normal value < 1 IU/mL, positive > 1.5–1.75 IU/mL, in bold = increased levels; ^ TRAb levels were measured during 2nd or 3rd trimester of pregnancy; postP = postpartum; ATD = antithyroid drugs; M = methymazole (mg/day); P = propylthyouracil (mg/day), - = no treatment, LT4 = levothyroxine (mcg/day); the recommended treatment dose is noted at each time frame; N = normal thyroid function, H = overt hyperthyroidism, SH = subclinical hyperthyroidism (TSH < 0.1 mIU/L, normal FT4 or/and FT3), hT = hypothyroxinemia (normal TSH, low FT4); Sh = subclinical hypothyroidism (TSH > 2.5 mIU/L, normal FT4); w = weeks of pregnancy, PD = premature delivery; A = APGAR score; * Pregnancy after IVF with donated oocyte; ^&^ Intrauterine death at 40 weeks due to a true umbilical cord knot; ** this child had normal weight and fetal morphometric ultrasound evaluation at 35 weeks (last known data); n.a = data not available.

**Table 3:** Serum TSH, FT4, and the dosage of antithyroid drugs in women with hyperthyroidism treated during pregnancy.

	Serum TSH (mIU/L)			Serum FT4 (ng/dL)		
Women treated more than 6 months before pregnancy *(n = 20 pregnancies, 16 women)*						
	T1	T2	T3	T1	T2	T3
Mean value (range)	0.40 (nd–1.06)	0.61 (nd–2.12)	0.57 (nd–1.9)	1.09 (0.82–1.12)	1.03 (0.71–2.04)	0.92 (0.63–1.44)
At least once below normal	7/19 (37%)	6/17 (35%)	4/18 (22%)	0/17 (0%)	2/17 (11.7%)	2/17 (11.7%)
At least once above normal	0/19 (0%)	0/17 (0%)	0/19 (0%)	1/17(5.8%)	0/17 (0%)	0/17 (0%)
Methimazole mg/day (median, range)	5 (2.5 at 2 days–10)	4.37 (2.5 at 2 days–10)	3.75 (2.5–10)			
	*n* *= 18*	*n* *= 6*	*n* *= 4*	-	-	-
Propylthiouracil mg/day (median, range)	150 (50–300)	75 (25–150)	75 (12.5–150)			
	*n* *= 5*	*n* *= 6*	*n* *= 5*	-	-	-
No treatment by the end of trimester	7/20 (35%)	12/20 (60%)	11/20 (55%)	-	-	-
**Women who started treatment shortly before (<6 weeks) or during pregnancy *(n = 8 pregnancies, 7 women)***						
Mean value (range)	nd	nd	0.24 (nd–1.2)	1.96 (1.3–2.63)	2.43 (1.18–5)	1.07 (0.59–1.73)
At least once below normal	3/3 (100%)	6/6 (100%)	4/6 (80%)	0/3	0/6	1/5 (20%)
At least once above normal	0/3 (0%) *p* *= 0.032*	0/6 (0%) *p* *= 0.013*	0/6 (0%) *p* *= 0.12*	2/3 (66%) *p* *= 0.045*	5/6 (83%) *p* *< 0.001*	1/5 (20%) *p* *= 0.22*
Methimazole mg/day (median, range)	20 (10–30) *n* *= 3*	10 (5–45) *n* *= 6*	5 (2.5/2 days–30) *n* *= 5*	-	-	-
Propylthiouracil	-	-	-	-	-	-
No treatment by the end of trimester	0/3 (0%) *p* *= 0.52*	0/6 (0%) *p* *= 0.017*	3/7 * (42.8%) *p* *= 1*	-	-	-

**Legend:** Below normal: TSH < 0.1 mIU/L, FT4 below the lower normal range of the assay; above normal: TSH > 2.5–3.5–3.5 mIU/L (in trimester 1—T1, 2—T2 and 3—T3, respectively), FT4 above the upper limit of the normal range of the assay; nd = not detectable (below the lower detection limit of the assay); * 1 patient, treated for only 1 month before pregnancy, had a spontaneous abortion at 5 weeks; 1 patient had a premature delivery at 32 weeks; *p* were calculated for comparison of the 2 groups of women.

**Table 4:** Pregnancy outcome.

KERRYPNX	Women with Hyperthyroidism (Previous or Current) *n* = 29 Women, 36 Single Fetus Pregnancies	Control Normal Women *n* = 39 Women, 39 Single Fetus Pregnancies	*p* Value
Age at pregnancy, years, mean ± SD	30.3 ± 4.7 29.6 ± 4.5 in ATD treated women	27.0 ± 4.1	<0.01
Pregnancy outcome	31 live children (91.1% of 34 evaluated; 2 unknown *) Women treated for more than 6 months before pregnancy (20 pregnancies): 1 fetal death at 40 weeks due to a true umbilical cord knot Women who started ATD shortly before or during pregnancy (8 pregnancies): 1 spontaneous abortion at 5 weeks 1 premature delivery at 32 weeks, with neonate death within 24 h (respiratory distress) Women in GD remission before pregnancy (5 pregnancies): 5 live children (1 unknown) Untreated hyperthyroidism (3 pregnancies): 1 live child (2 unknown *)	39 live children	
Delivery type	Vaginal: 10 (28.5%) C-section: 23 (65.7%); 85% in women treated >6 months; 57% in women diagnosed shortly before or during pregnancy, *p* = 0.049 Unknown: 2 (5.7%)	Vaginal: 17 (43.5%) C-section: 22 (56.5%)	0.32
Mean gestational age (weeks)	38.4 ± 1.7 38.5 in women treated > 6 months, 38.0 in women treated shortly before or during pregnancy (*p* = NS)	39.05 ± 1.2	0.11
Children Sex F:M Congenital anomalies Follow-up >2 years Fetal/neonatal hyperthyroidism Fetal/neonatal goiter or congenital hypothyroidism	*n* = 35 *	*n* = 39	
	20 F:11 M (4 unknown)	15 F:24 M	0.053
	1 small atrial sept defect—ostium secundum (in 25 live children of ATD treated women, 4%)	None	
	13 of 25 children of ATD treated women (52%)	N/A	
	1 child (in 30 live children from GD women, 3.3%)	0 cases	
	0 cases	0 cases	
Children’s birth weight, mean ± SD, grams	(a) All women treated during pregnancy (*n* = 24 children): 3267 ± 515	3342 ± 469	0.56
	(b) Women treated more than 6 months before, and during pregnancy (*n* = 18 children): 3238 ± 480		0.45
	(c) Women who stopped ATD before week 10 (*n* = 6 children): 3208 ± 391		0.47
	(d) Women diagnosed and treated shortly before (*n* = 1) or only during pregnancy (*n* = 5): 3352 ± 652 *p* = NS between groups (b), (c), (d)		0.97

Legend. GD = Graves’ disease; TRAB = Thyrotropin receptor antibodies; F = female; M = male, n.a. = not available; * 1 child had female sex, normal weight and normal fetal morphometric ultrasound evaluation at 35 weeks (last known data).

## Data Availability

The data supporting the results presented in this study are available on request from the corresponding author. The data are not publicly available due to ethical reasons.
